# Supplementary figures and images for: A signature of Neanderthal introgression on molecular mechanisms of environmental responses
Source: PLoS Genet. 2021 Sep 27;17(9):e1009493. doi: 10.1371/journal.pgen.1009493 (PMC8509894; doi:10.1371/journal.pgen.1009493)

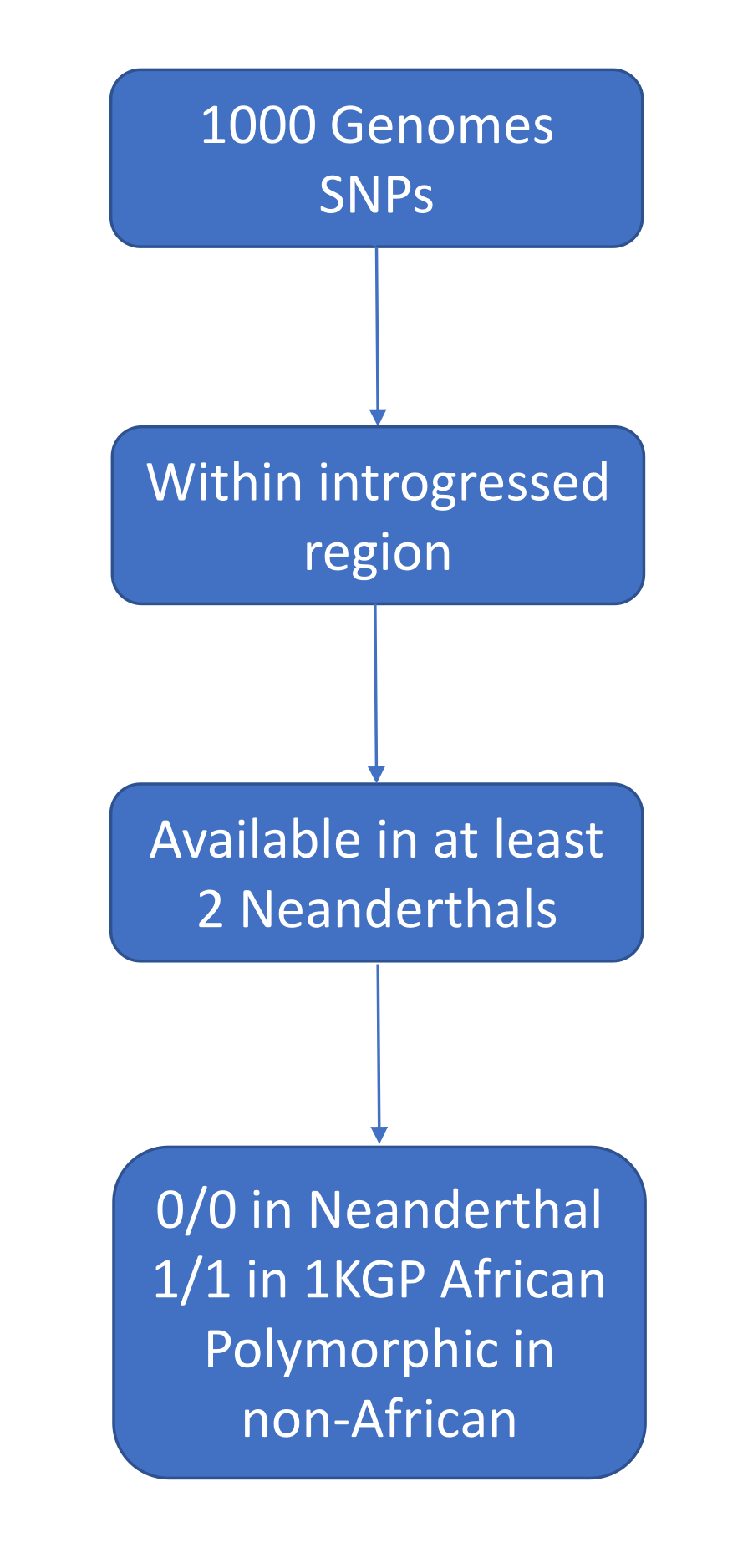

Supplement: S1 Fig — (TIF) [file pgen.1009493.s007.tif]

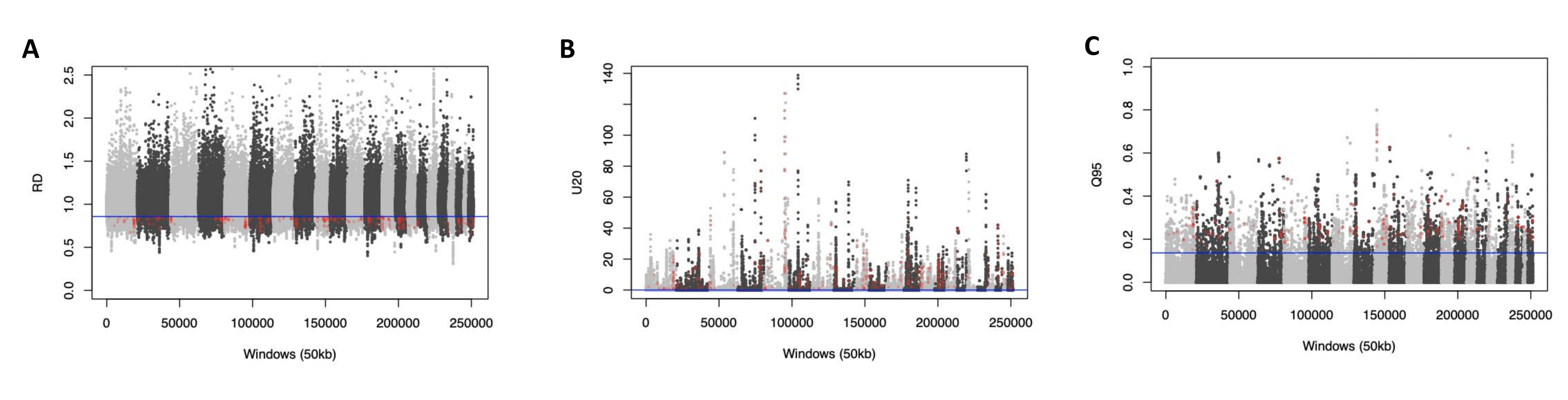

Supplement: S2 Fig — Adaptive introgression statistics (A) RD, (B) U20, and (C) Q95 statistics for 50kb windows genome-wide. The horizontal line indicates the 5th percentile, and windows with p<0.05 with a N-eQTL are colored red. (TIF) [file pgen.1009493.s008.tif]

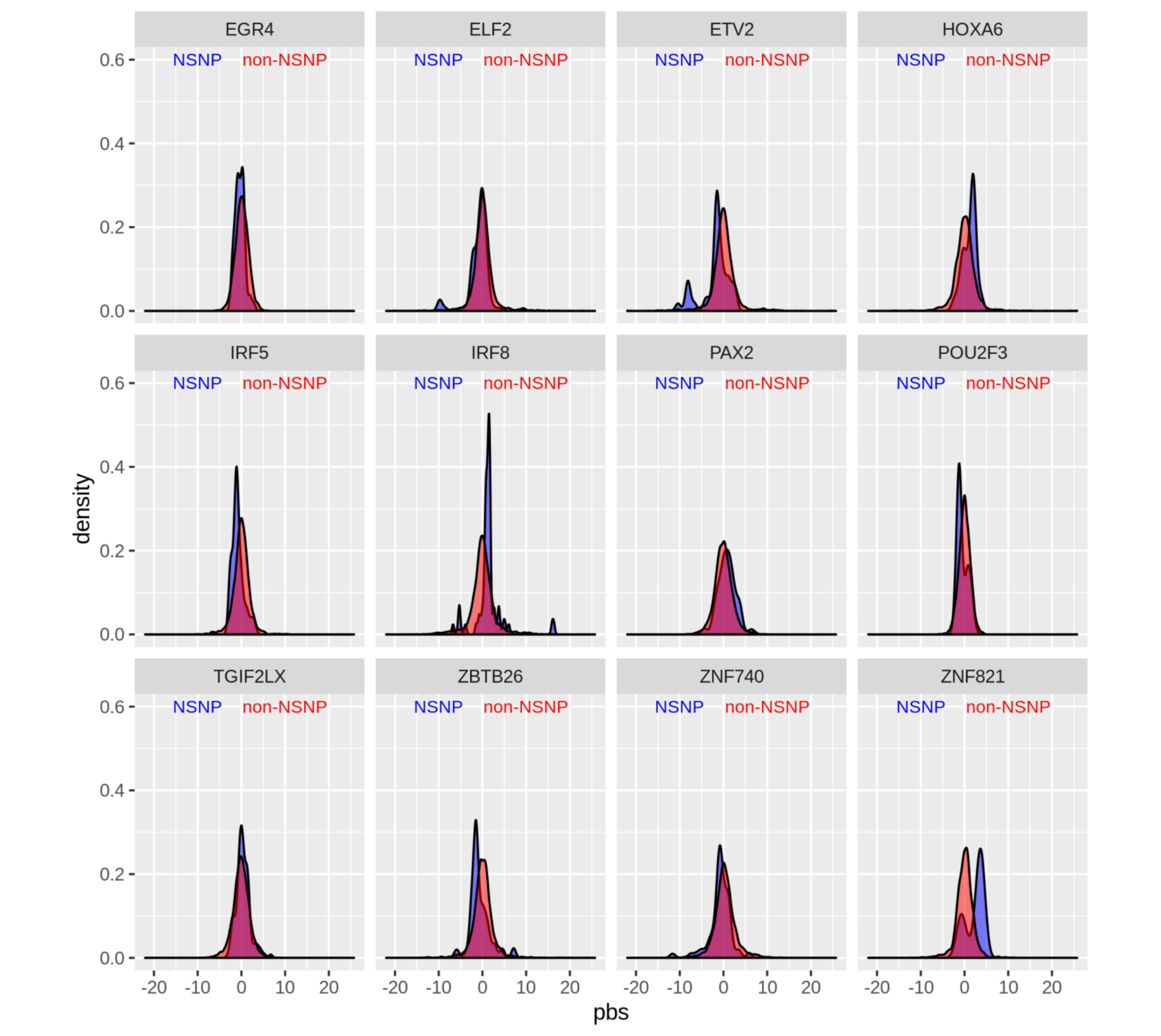

Supplement: S3 Fig — Density plots for significant Kolmogorov-Smirnov test results comparing the distribution of PBS values for N-SNPs and non-N-SNPs for individual transcription factors. The blue color represents N-SNPs and the red represents non-N-SNPs. (TIF) [file pgen.1009493.s009.tif]

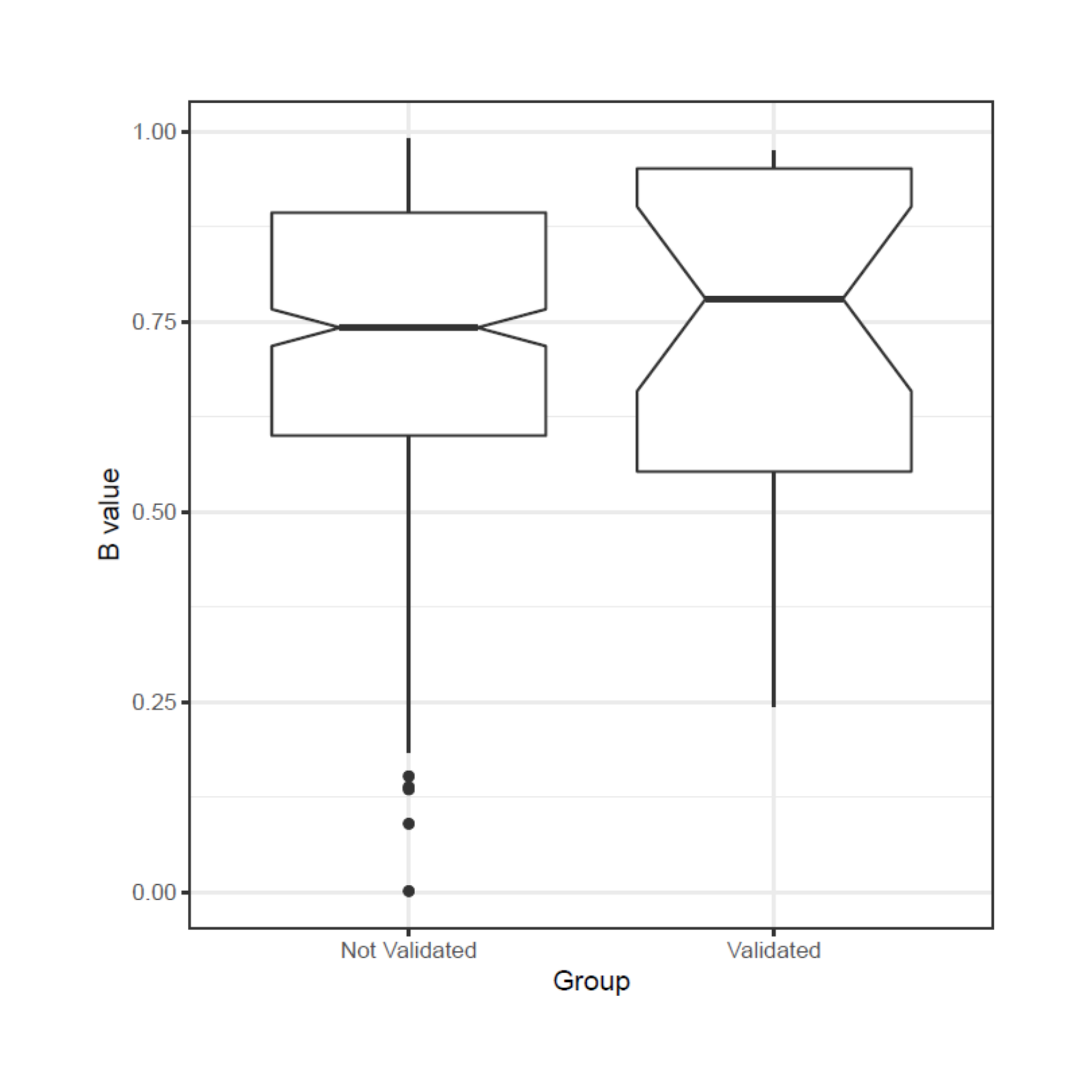

Supplement: S4 Fig — (TIF) [file pgen.1009493.s010.tif]

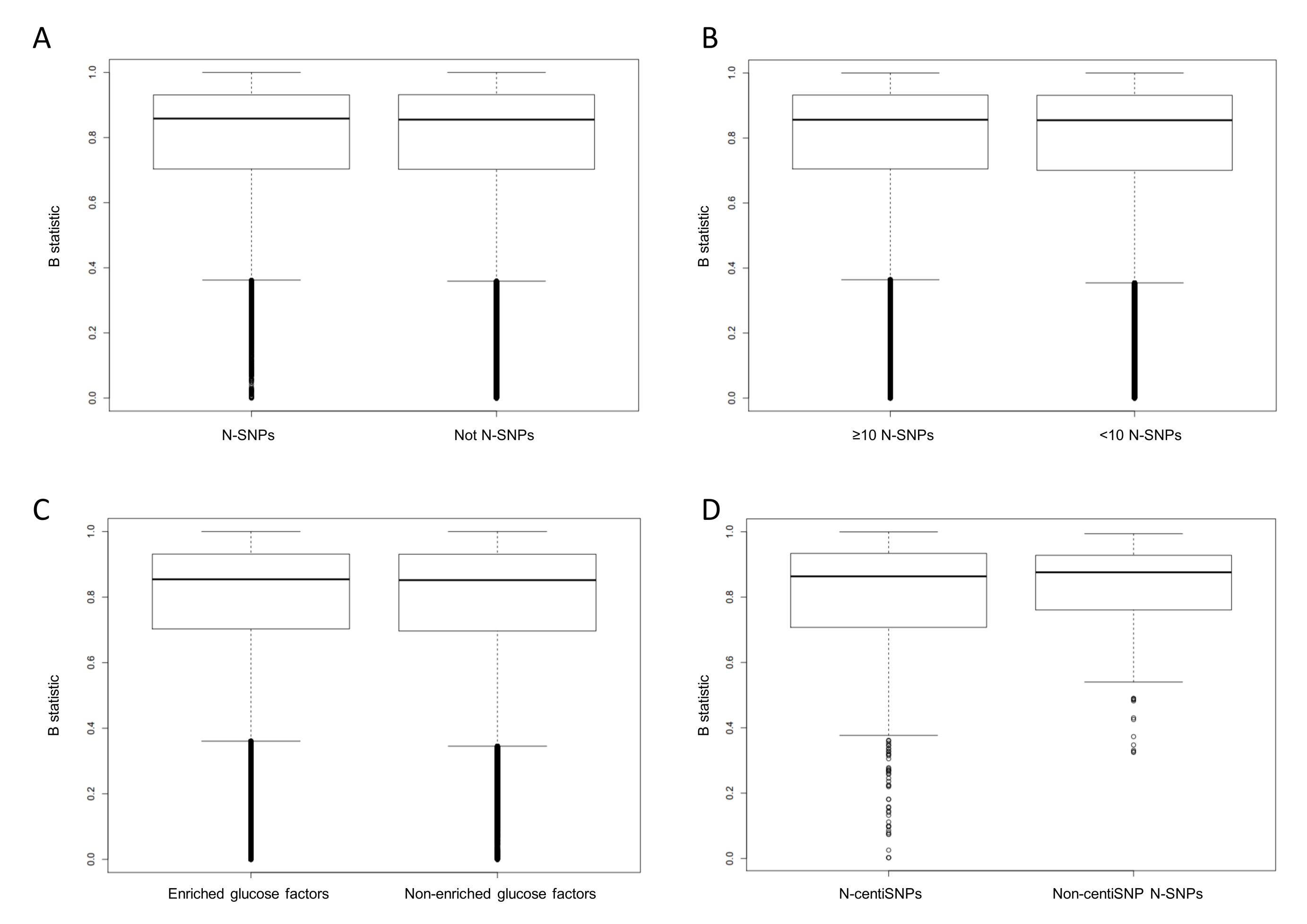

Supplement: S5 Fig — B-statistic comparisons (A) Distribution of B statistics for centiSNPs which are N-SNPs and centiSNPs which are not N-SNPs. (B) Distribution of B statistics forSNPs in TF footprints with > = 10 N-SNPs in footprint and for SNPs in TF footprints with < 10 N-SNPs in footprint. (C) Distribution of B statistics for centiSNPs in 16 TF footprints relevant for glucose metabolism that are enriched for N-centiSNPs and for a control set of centiSNPs in 21 TF footprints relevant for glucose metabolism but that are not enriched for N-centiSNPs. (D) Distribution of B statistics for N-centiSNPs and non-centi N-SNPs in footprints for the most enriched TFs (ARNT, SWI5, AML1, AP1, c-Jun, ATF2, Elk1). (TIF) [file pgen.1009493.s011.tif]

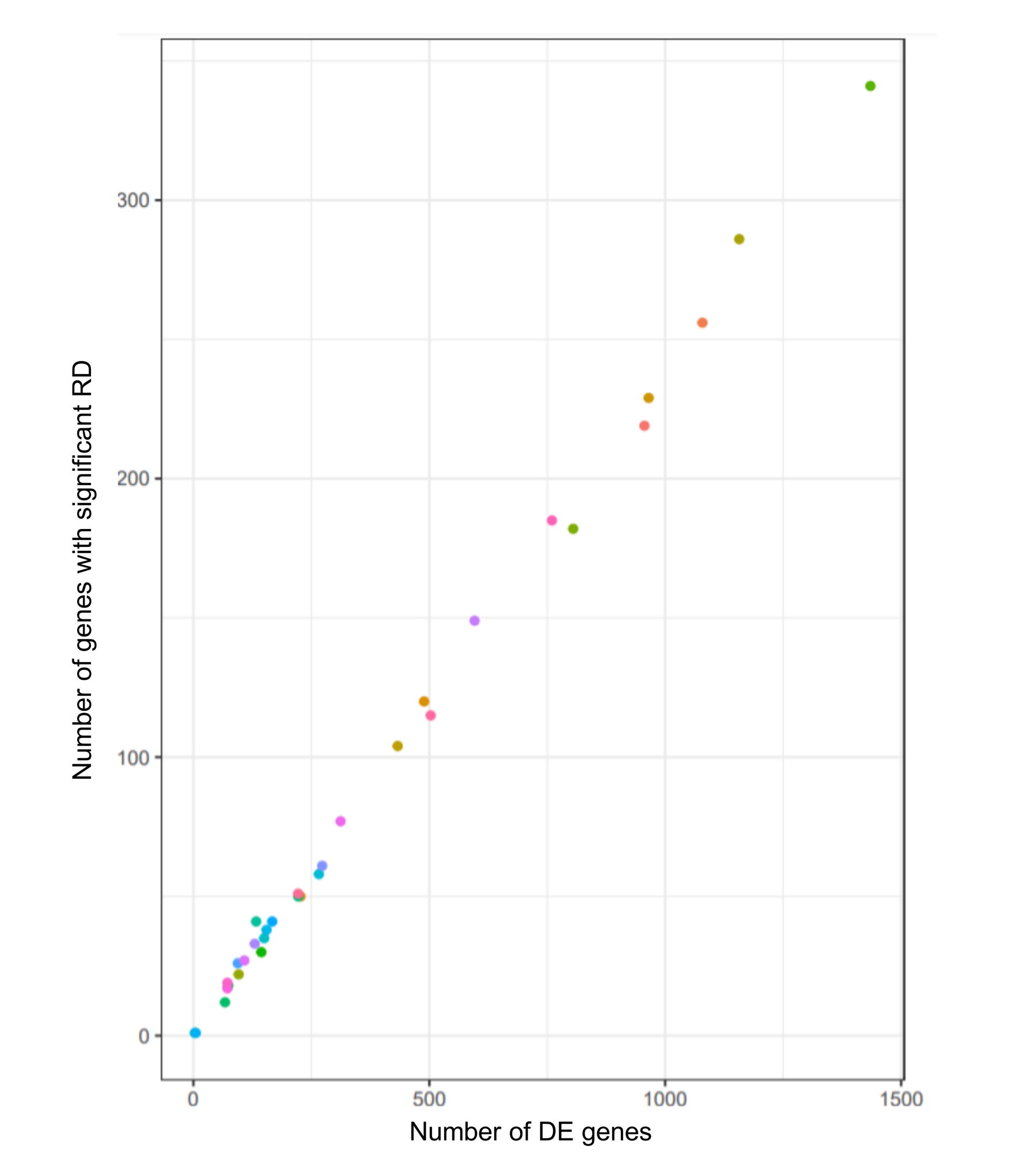

Supplement: S6 Fig — The x-axis represents the number of DEGs per treatment, and the y-axis represents the number of genes in regions with evidence of adaptive introgression from the RD statistics. Each dot represents a different treatment. (TIF) [file pgen.1009493.s012.tif]
